# Pioglitazone, a Peroxisome Proliferator-Activated Receptor γ Agonist, Suppresses Rat Prostate Carcinogenesis

**DOI:** 10.3390/ijms17122071

**Published:** 2016-12-10

**Authors:** Shugo Suzuki, Yukiko Mori, Aya Nagano, Aya Naiki-Ito, Hiroyuki Kato, Yuko Nagayasu, Mizuho Kobayashi, Toshiya Kuno, Satoru Takahashi

**Affiliations:** 1 Department of Experimental Pathology and Tumor Biology, Nagoya City University Graduate School of Medical Sciences, Nagoya 467-8601, Japan; ymori345@gmail.com (Y.M.); me_me_sheep3@yahoo.co.jp (A.N.); ayaito@med.nagoya-cu.ac.jp (A.N.-I.); h.kato@med.nagoya-cu.ac.jp (H.K.); naga-p@dk.pdx.ne.jp (Y.N.); k-miz@topaz.ocn.ne.jp (M.K.); tkuno@med.nagoya-cu.ac.jp (T.K.); sattak@med.nagoya-cu.ac.jp (S.T.); 2 Pathology Division, Nagoya City East Medical Center, Nagoya 464-8547, Japan

**Keywords:** peroxisome proliferator-activated receptor γ, pioglitazone, prostate, carcinogenesis, prostate cancer, nuclear factor κB

## Abstract

Pioglitazone (PGZ), a peroxisome proliferator-activated receptor γ agonist, which is known as a type 2 diabetes drug, inhibits cell proliferation in various cancer cell lines, including prostate carcinomas. This study focused on the effect of PGZ on prostate carcinogenesis using a transgenic rat for an adenocarcinoma of prostate (TRAP) model. Adenocarcinoma lesions as a percentage of overall lesions in the ventral prostate were significantly reduced by PGZ treatment in a dose-dependent manner. The number of adenocarcinomas per given area in the ventral prostate was also significantly reduced by PGZ treatment. The Ki67 labeling index in the ventral prostate was also significantly reduced by PGZ. Decreased cyclin D1 expression in addition to the inactivation of both p38 mitogen-activated protein kinase (MAPK) and nuclear factor (NF)κB were detected in PGZ-treated TRAP rat groups. In LNCaP, a human androgen-dependent prostate cancer cell line, PGZ also inhibited cyclin D1 expression and the activation of both p38 MAPK and NFκB. The suppression of cultured cell growth was mainly regulated by the NFκB pathway as detected using specific inhibitors in both LNCaP and PC3, a human androgen-independent prostate cancer cell line. These data suggest that PGZ possesses a chemopreventive potential for prostate cancer.

## 1. Introduction

Prostate cancer is the second most commonly-diagnosed cancer in males around the world, including in economically-developed countries [1], with its frequency rapidly increasing in Japan [2]. Androgen ablation therapy is widely used as treatment during the initial stages of this disease when it may produce a favorable outcome, but in most cases, the therapy eventually fails, and death occurs from recurrent androgen-independent prostate cancer and metastasis. Therefore, efforts need to be directed toward developing novel strategies, such as chemoprevention, to reverse, suppress, prevent or delay the progression of prostate cancer. In the field of chemoprevention, large prospective randomized trials, such as the Prostate Cancer Prevention Trial (PCPT), the Reduction by Dutasteride of Prostate Cancer Events (REDUCE) trial and the Selenium and Vitamin E Cancer Prevention Trial (SELECT), have been undertaken. However, their findings have not been approved for chemoprevention by the U.S. Food and Drug Administration (FDA) [3,4]. Therefore, the search for an appropriate chemopreventive agent for prostate cancer is ongoing.

Peroxisome proliferator-activated receptor γ (PPARγ) is a ligand-activated transcription factor that belongs to the class of nuclear receptors. It induces adipocyte differentiation, is involved in the control of inflammatory reactions and improves insulin sensitivity in glucose metabolism [5,6]. Recently, PPARγ agonists have been shown to inhibit cell proliferation or induce apoptosis in various cancer cell lines, including prostate carcinomas [5,6,7,8]. In in vivo studies, PPARγ agonists also inhibited colon, tongue and liver carcinogenesis [9,10,11]. Additionally, PPARγ heterozygous (+/−) mice display a higher susceptibility to 7,12-dimethylbenz[*a*]anthracene (DMBA)-induced mammary, ovarian and skin carcinogenesis compared to the wild-type [12]. Meanwhile, PPARγ and dual PPARα/γ agonists increased the incidence of bladder cancer in rats as determined by two-year bioassays, but this is not apparent in mice [13].

In this study, we investigated the inhibitory effects of pioglitazone (PGZ), a PPARγ agonist, on prostate carcinogenesis in the transgenic rat in an adenocarcinoma of prostate (TRAP) model that was generated in our laboratory. This model features the development of well-differentiated prostate adenocarcinomas in prostatic lobes within a short period of time [14,15].

## 2. Results

### 2.1. PGZ Inhibited the Progression of Prostate Tumorigenesis as Well as Cell Proliferation in TRAP Rats

Significant differences in final body, liver, kidneys and spleen weights among the groups were not noted, but the weight of the ventral prostate in 5 mg/kg PGZ-treated rats was significantly lower than that in control rats (Table 1; *p* < 0.05). Changes indicative of toxicity in rat liver and kidneys in response to PGZ treatment were not noted as determined by assessing histological sections (Figure S1).

Serum testosterone levels tended to be reduced by PGZ in a dose-dependent manner, but this was not significant (control, 1 and 5 mg/kg PGZ: 5.5 ± 3.2, 3.7 ± 3.1 and 2.8 ± 2.2 ng/mL, respectively). The serum level of estradiol was not significantly affected by PGZ (control, 1 and 5 mg/kg PGZ: 20.7 ± 3.7, 20.8 ± 4.5 and 21.4 ± 4.2 pg/mL, respectively).

**Figure 1:**
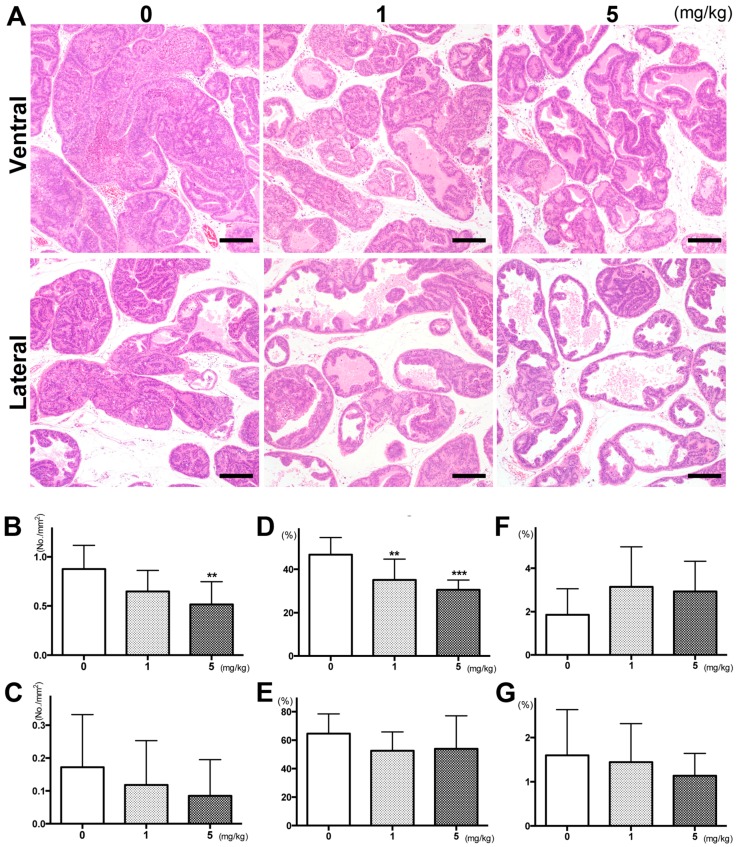
Effects of pioglitazone (PGZ) on prostate lesions. Representative histopathological findings for lesions in the ventral and lateral prostates of the 0 (control), 1 and 5 mg/kg PGZ-treated groups (**A**), Scale bars = 200 µm; The number of adenocarcinoma lesions per area in ventral (**B**) and lateral (**C**) prostates of transgenic rats in an adenocarcinoma of prostate (TRAP) rat model treated with PGZ. Ki67 and TUNEL labeling indices in ventral (**D**,**F**) and lateral (**E**,**G**) prostates of TRAP rats treated with PGZ, respectively. Data are the mean ± SD values from more than three independent experiments. ** *p* < 0.01, *** *p* < 0.001 compared to the untreated control (0 mg/kg), respectively.

Representative histopathological findings of ventral and lateral prostates in each group are presented in Figure 1A. In the ventral prostate of TRAP rats, there was a marked or partial response to PGZ treatment as demonstrated by a significant reduction in prostate neoplastic lesions; however, small foci still remained. A decrease in the incidence of adenocarcinoma was also observed in the lateral prostate in response to PGZ treatment (Table 2). In the ventral prostate, a quantitative evaluation of the proportion of preneoplastic and neoplastic lesions in the prostate gland showed a significant suppression of progression from low grade prostatic intraepithelial neoplasia (LG-PIN) or high grade PIN (HG-PIN) to adenocarcinoma in rats treated with PGZ in a dose-dependent manner (Table 2; *p* < 0.01 for 1 mg/kg PGZ and *p* < 0.001 for 5 mg/kg PGZ). With regard to adenocarcinoma, the number of foci per area in the ventral prostate was significantly reduced by 5 mg/kg PGZ (Figure 1B; *p* < 0.01). Meanwhile, in the lateral prostate, the suppression of progression and the number of adenocarcinomas per area tended to be reduced by PGZ, but not significantly (Table 2 and Figure 1C, respectively). A difference in the average size of adenocarcinomas in both ventral and lateral prostates was not noted among the groups (ventral prostate: control, 1 and 5 mg/kg PGZ: 0.10 ± 0.02, 0.08 ± 0.02 and 0.09 ± 0.01 mm^2^; lateral prostate: control, 1 and 5 mg/kg PGZ: 0.06 ± 0.06, 0.04 ± 0.04 and 0.05 ± 0.05 mm^2^, respectively). The size of adenocarcinoma in the ventral prostate inversely correlated with the PGZ dose, but this was not significant (ρ = −0.33, *p* < 0.09).

There was a significant decrease, in a dose-dependent manner, in the labeling index of Ki-67 in HG-PIN of the ventral prostate of TRAP rats given PGZ (Figure 1D; *p* < 0.01 for 1 mg/kg, *p* < 0.001 for 5 mg/kg), but not in the lateral prostate (Figure 1E). Differences in the labeling index of TUNEL in both ventral and lateral prostates among the groups were not noted (Figure 1F,G, respectively). Androgen receptor (AR) and SV40 T antigen were detected immunohistochemically in a diffuse pattern in areas of PIN and adenocarcinomas in the ventral prostate, with differences not observed among the groups (Figure S2).

In summary, PGZ treatment of TRAP rats significantly reduced the number of adenocarcinomas per area, the progression from LG-PIN or HG-PIN to adenocarcinoma and cell proliferation as determined by the Ki67 labeling index in HG-PIN within the ventral prostate in a dose-dependent manner. PGZ treatment of TRAP rats did not affect apoptosis nor the distribution of AR or SV40 T antigen.

### 2.2. Reduction of Cell Proliferation by PGZ Treatment Was Associated with Changes in the NFκB and p38 Pathways

**Figure 2:**
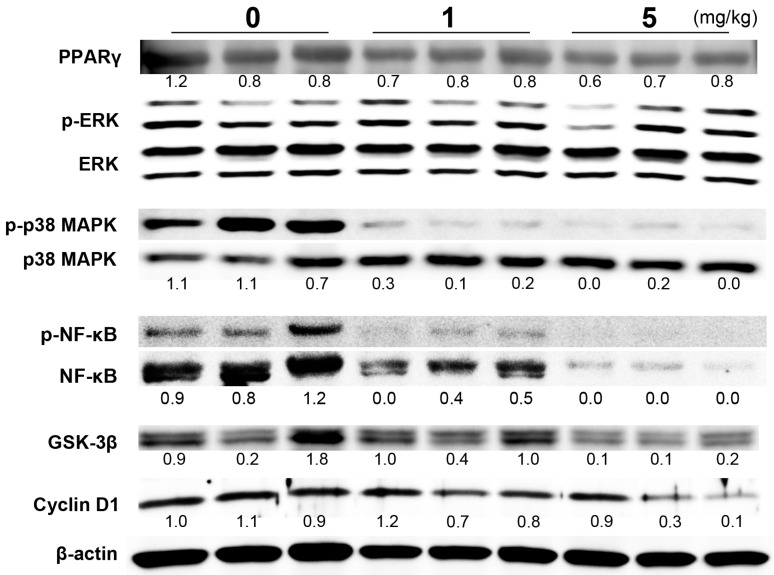
Immunoblot analysis in ventral prostate. Representative immunoblots of PPARγ, phospho-ERK (p-ERK), ERK, phospho-p38 MAPK (p-p38 MAPK), p38 MAPK, phospho-NFκB (p-NFκB), NFκB, GSK-3β and cyclin D1 in ventral prostate samples of TRAP rats treated with PGZ.

Previous studies have shown that mitogen-activated protein kinases (MAPKs) became inactivated after the treatment of cancer cells with chemopreventive agents, such as angiotensin II receptor blocker, purple corn color or apocynin [16,17,18]. To elucidate the mechanisms of anti-carcinogenesis by PGZ, we therefore investigated the activation of p38 MAPK and ERK1/2 in prostate tissues of TRAP rats. In this study, a reduction in phosphorylation of p38 MAPK was detected in the ventral prostates of PGZ-treated rats; ERK1/2 remained unchanged (Figure 2). The expression of glycogen synthase kinase (GSK)-3β and the phosphorylation of nuclear factor (NF)κB were also reduced in the ventral prostate of PGZ-treated rats (Figure 2), for which regulation has been reported in LNCaP cells by the PPARγ agonist, troglitazone [8]. A reduction of cyclin D1 protein was detected in PGZ-treated rats in parallel with a decreased Ki-67 index (Figure 2). Meanwhile, PPARγ expression was similar among all groups (Figure 2).

Therefore, PGZ treatment of TRAP rats inactivated p38 MAPK and NFκB and reduced the expression of GSK-3β and cyclin D1.

### 2.3. PGZ Affected the Reduction of Cell Growth in Human Prostate Cancer Cell Lines

**Figure 3:**
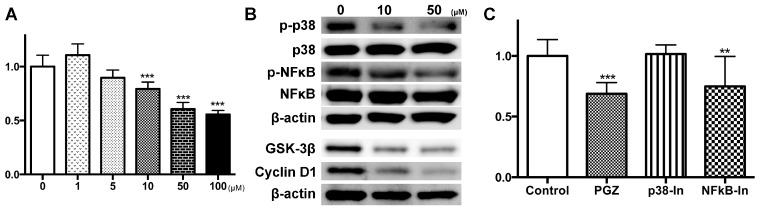
Anti-growth effects of PGZ in LNCaP cells. LNCaP cells were incubated with PGZ for 72 h, and then, cytotoxicity was assessed by the WST-1 assay (**A**); Representative immunoblots of phospho-p38 MAPK (p-P38 MAPK), p38 MAPK, phospho-NF-κB (p-NFκB), NFκB, GSK-3β and cyclin D1 in LNCaP cells (**B**); The effect of a specific p38 MAPK inhibitor (p38-In) and an NFκB activation inhibitor (NFκB-In) on LNCaP cell proliferation (**C**). Results are expressed as a proportion of untreated control (**A**,**C**). Data are expressed as the mean ± SD and are from more than three independent experiments (**A**,**C**). ** *p* < 0.01, *** *p* < 0.001 compared to the untreated control (0 µM), respectively.

**Figure 4:**
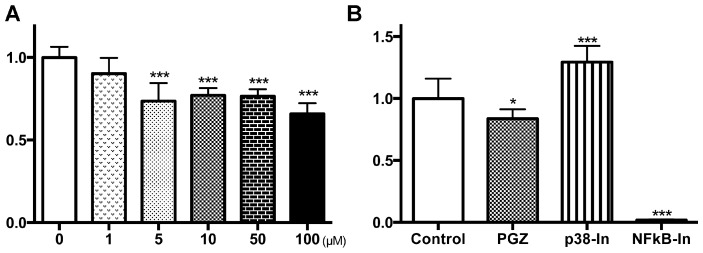
Anti-growth effects of PGZ in PC3 cells. PC3 cells were incubated with PGZ for 72 h, and then cytotoxicity was assessed by the WST-1 assay (**A**); The effect of a specific p38 MAPK inhibitor (p38-In) and an NFκB activation inhibitor (NFκB-In) on PC3 cell proliferation (**B**). Results are expressed as a proportion of the untreated control. Data are expressed as the mean ± SD and are from more than three independent experiments. * *p* < 0.05, *** *p* < 0.001 compared to the untreated control (0 µM).

In LNCaP and PC3 cells, a significant reduction in cell growth was detected after PGZ treatment at concentrations higher than 10 and 5 µM, respectively (Figure 3A and Figure 4A, respectively; *p* < 0.001 for all). Expressions of p-p38 MAPK, p-NFκB, GSK-3β and cyclin D1 proteins were reduced by PGZ treatment in LNCaP cells (Figure 3B). Cell growth of LNCaP was significantly suppressed by using an NFκB inhibitor (*p* < 0.01), but not a p38 MAPK inhibitor (Figure 3C). Cell growth of PC3 was also significantly suppressed by using an NFκB inhibitor (*p* < 0.001), but significantly promoted by using a p38 MAPK inhibitor (*p* < 0.001; Figure 4B).

Thus, PGZ treatment of both LNCaP and PC3 prostate cells reduced cell growth mainly via the inactivation of the NFκB pathway.

## 3. Discussion

In the present study, we demonstrated the suppressive effects of PGZ on prostate carcinogenesis in the TRAP rat model. We detected not only a reduction of carcinoma, but also an increase of LG-PIN. In TRAP rats, carcinomas are thought to develop from LG-PIN through to HG-PIN as an intermediate stage. We postulated that some lesions were inhibited in developing from HG-PIN to carcinoma, while other lesions were inhibited in changing from LG-PIN to HG-PIN. This may explain why we previously detected an increase in LG-PIN and a decrease of carcinoma in the ventral prostate in TRAP rats treated with apocynin. Meanwhile, there were fewer differences in HG-PIN per given area among the groups.

A reduction of cancer in the lateral prostate was also detected, but this was not significantly different among treatment and control groups. This may have been due to the small number of carcinomas observed, which would have made it difficult to observe any significant difference in cancer within the lateral prostate. The effects of PGZ on Ki67 in the lateral prostate were less than those observed in the ventral prostate. Gene expression analysis in the rodent prostate indicated that the gene expression pattern differed among the lobes [19]. For example, some prostate carcinogens, such as 2-amino-1-methyl-6-phenylimidazo[4,5-*b*]pyridine (PhIP) and 3,2′-dimethyl-4-aminobiphenyl (DMAB), are known to induce prostate cancer in the ventral, but not lateral prostate [20,21]. In the same vein, we thought that the effects of PGZ in the ventral prostate were greater than in the lateral prostate in this study.

In an in vitro study, PPARγ agonists inhibited cell proliferation in prostate carcinomas [6,8], but the effects of PPARγ agonists in an in vivo carcinogenesis model were less well described, especially for prostate carcinogenesis. The PGZ dose of 1 mg/kg used in this study is approximately the same dose used for type 2 diabetic patients. Toxic effects with regard to body weights or histological changes in liver and kidneys of both 1 and 5 mg/kg PGZ-treated rats were not observed in this study. The reduction in ventral prostate weight may be reflected by the proportion of the carcinoma area because the central area of the carcinoma was occupied by tight atypical cells compared to PIN, especially LG-PIN. It may also be reflected by the size of the adenocarcinoma, but this was not statistically different. Overall, our data suggest a chemopreventive effect of PGZ on the development of human prostate cancer.

With regard to the relationship between animal models and human cohort or clinical case studies involving PGZ, the suppression of colorectal and liver carcinogenesis by PGZ was initially described in an animal model [9,11]. A statistically-significant reduction of cancer risks by the use of thiazolidinediones, including PGZ, was also described in a recent meta-analysis [22].

However, PGZ has also been reported to induce urothelial bladder tumors in rats [23], while cohort and nested case-control analyses indicated an increased risk of bladder cancer with PGZ use [22,24]. In a study of the mechanism of bladder carcinogenesis by PGZ treatment in rats, the induction of tumors was related to the increased formation of urinary solids, but was not detected in mice [13,25]. After a multi-population pooled cumulative exposure analysis in humans, however, a relationship between exposure to PGZ and bladder cancer risk was conclusively not found [26]. In prostate cancer, there is thought to be less of a relationship between the use of thiazolidinediones and cancer risk [22,27].

Thiazolidinediones were introduced to the market in the late 1990s, and the mean age of onset of prostate cancer is generally later than that of other cancers [2,28]. For these reasons, it may therefore presently be premature to investigate the chemopreventive effects of PGZ on prostate cancer development in humans by epidemiological studies.

With regard to the effects of PGZ in prostate carcinogenesis, we found the inhibition of cell proliferation, but not the induction of apoptosis, in prostates of PGZ-treated rats. To check the regulation of cell proliferation by PGZ, we assessed the reduction of phosphorylation in p38 MAPK and NFκB in both rat prostate tumor tissue and in a human prostate cancer cell line, LNCaP. Using specific inhibitors, NFκB signaling was found to be the main pathway for cell proliferation, at least in both castration-sensitive and -resistant human prostate cancers. These results indicate that PGZ has the ability to not only display anti-cancer properties, but may also have an anti-carcinogenic potential in prostate cancer via the NFκB pathway. Meanwhile, the different effects observed with a p38 MAPK inhibitor on LNCaP and PC3 cell proliferation may be related to androgen independency.

Recently, telmisartan, as a PPAR-gamma agonist, was found to inhibit prostate cancer via the induction of apoptosis [29]; it also inhibited prostate carcinogenesis [16]. Our findings thus suggest that the regulation of PPAR may become an important pathway to counteract the development of prostate cancer.

## 4. Materials and Methods

### 4.1. Animal Experiments

Male heterozygous TRAP rats with a Sprague–Dawley (SD) genetic background that were established in our laboratory were used in the present study. All animals were housed in plastic cages on wood-chip bedding in an air-conditioned, specific pathogen-free animal room at 22 ± 2 °C and 55% ± 5% humidity with a 12-h light/dark cycle and fed a basal diet (Oriental MF, Oriental Yeast Co., Tokyo, Japan) and water, ad libitum. All animal experiments were performed under the protocols approved by the Institutional Animal Care and Use Committee of Nagoya City University Graduate School of Medical Sciences (Code: H25M_41, 7 November 2013).

Six-week-old TRAP rats were divided into three groups; the animals were gavaged with PGZ (0, 1 and 5 mg/kg; Tokyo Chemical Industry Co., Ltd., Tokyo, Japan) five times per week, and body weights were estimated weekly. At experimental Week 8, under deep isoflurane anesthesia, blood was collected between 9 and 11 a.m. and testosterone and estradiol levels measured using radioimmunoassays by a commercial laboratory (The Tohkai Cytopathology Institute, Gifu, Japan). The urogenital complex of each rat was removed as a whole, together with the seminal vesicles, and then, the ventral prostate was weighed. A portion of each prostate gland was immediately frozen in liquid nitrogen and stored at −80 °C until being processed. After this, the remaining prostate tissue, liver, kidneys and spleen were fixed in 10% formalin. The tissues were routinely processed to paraffin-embedded sections and stained with hematoxylin and eosin (H&E).

### 4.2. Histopathology, Immunohistochemistry and TUNEL Assays

Neoplastic lesions in the prostate gland of TRAP rats were evaluated as previously described [17,18,30]. Briefly, neoplastic lesions were classified into three types: LG-PIN, HG-PIN and adenocarcinoma. The relative numbers of acini with the histological characteristics of each type, that is LG-PIN, HG-PIN and adenocarcinoma, were quantified by counting the total acini in each prostatic lobe. Deparaffinized sections were incubated with diluted antibodies for rabbit monoclonal Ki67 (Abcam plc, Cambridge, UK), rabbit polyclonal AR and SV40 T antigen (Santa Cruz Biotechnology Inc., Dallas, TX, USA). Apoptotic cells in the prostate were detected using an In Situ Apoptosis Detection Kit (TUNEL method) according to the manufacturer’s instructions (Takara Bio Inc., Ohtsu, Japan). The number of Ki67- or TUNEL-labeled cells in at least 1000 cells was counted to determine labeling indices of HG-PIN.

### 4.3. Cell Lines

The androgen-dependent and -independent human prostate cancer cell lines, LNCaP and PC3, respectively, were purchased from The American Type Culture Collection (ATCC; Manassas, VA, USA). LNCaP and PC3 cells were cultured in Roswell Park Memorial Institute 1640 Medium (RPMI 1640, Gibco, Life Technologies, Carlsbad, CA, USA) with 10% fetal bovine serum (FBS; Thermo Fisher Scientific Inc., Waltham, MA, USA) in a humidified incubator with an atmosphere comprising 95% air and 5% CO_2_ at 37 °C.

### 4.4. Cell Proliferation Assay

The proliferation of LNCaP or PC3 cells was assessed by the 4-[3-(4-iodophenyl)-2-(4-nitrophenyl)-2*H*-5-tetrazolio]-1,3-benzene disulfonate tetrazolium salt (WST-1) assay (Roche Applied Science, Mannheim, Germany). Briefly, cells were seeded in 96-well plates at 2000 cells/well/100 µL of culture medium. PGZ (1 to 100 µM), a p38 MAPK inhibitor (5 µM, SB202190; EMD Millipore, Billerica, MA, USA) or a NFκB activation inhibitor (10 µM, BAY 11-7082; EMD Millipore) was added 24 h after seeding and cells incubated for a further three days. WST-1 reagent was added to each well incubated for 60 min at 37 °C, and then, the absorbance of each well was measured at 430 nm.

### 4.5. Immunoblot Analyses

Ventral prostate tissues or LNCaP cells were lysed on ice with radioimmunoprecipitation assay (RIPA) buffer (Pierce Biotechnology, Rockford, IL, USA) containing a protease inhibitor (Pierce Biotechnology). The insoluble matter was removed by centrifugation at 12,000 rpm for 20 min at 4 °C, and the supernatants were collected. Protein concentrations were determined with a Coomassie Plus-The Better Bradford Assay Kit (Pierce Biotechnology). Samples were mixed with sample buffer (Bio-Rad Laboratories, Hercules, CA, USA), heated for 5 min at 95 °C and then subjected to sodium dodecyl sulfate-polyacrylamide gel electrophoresis (SDS-PAGE). The separated proteins were transferred onto nitrocellulose membranes, followed by blocking with SuperBlock Blocking Buffer (Thermo Fisher Scientific Inc.) for 1 h at room temperature. Membranes were probed with antibodies for rabbit polyclonal PPARγ (Thermo Fisher Scientific Inc.), rabbit polyclonal cyclin D1, rabbit polyclonal p38 MAPK, rabbit monoclonal phospho-p38 (p-p38) MAPK, rabbit polyclonal NFκB-p65 (NFκB), rabbit polyclonal phospho-NFκB-p65 (p-NFκB), rabbit monoclonal GSK-3β, rabbit polyclonal p44/42 MAPK (ERK1/2) or rabbit polyclonal phospho-ERK1/2 (Cell Signaling Technology, Inc., Danvers, MA, USA) in 1× TBS with 0.1% Tween 20 at 4 °C overnight, followed by exposure to peroxidase-conjugated appropriate secondary antibodies and visualization with an enhanced chemiluminescence detection system (GE Healthcare Bio-Sciences, Buckinghamshire, UK). To confirm equal protein loading, each membrane was stripped and reprobed with mouse monoclonal anti-β-actin (Sigma-Aldrich, Co., St. Louis, MO, USA). All antibodies cross-react with both human and rat antigens.

### 4.6. Statistical Analysis

All in vitro experiments were performed at least in triplicate to confirm reproducibility. Statistical analyses were performed with the mean ± standard deviation (SD) values using one-way ANOVA and Dunnett’s test by Prism ver. 6 (GraphPad Software, Inc., La Jolla, CA, USA). *p* < 0.05 was considered statistically significant.

## 5. Conclusions

PGZ suppressed prostate carcinogenesis in the TRAP rat model. The mechanism of prevention appears to involve the regulation of cell proliferation via the NFκB pathway. PGZ therefore warrants further attention as a promising chemopreventive drug for prostate cancer.

## Figures and Tables

**Table 1:** Body and organ weights.

PGZ Treatment	No. of Rats	Body Weight (g)	Liver (g)	Kidneys (g)	Spleen (g)	Ventral Prostate (g)
Control	10	506.6 ± 54.3	15.9 ± 1.5	3.0 ± 0.2	0.8 ± 0.1	0.30 ± 0.07
1 mg/kg	8	531.9 ± 69.7	16.9 ± 2.6	3.0 ± 0.3	0.8 ± 0.2	0.27 ± 0.06
5 mg/kg	9	531.4 ± 25.7	16.2 ± 1.5	3.0 ± 0.2	0.8 ± 0.1	0.24 ± 0.04 *

Values are expressed as the mean ± SD; * *p* < 0.05 compared to the control group; PGZ, pioglitazone.

**Table 2:** Incidence of carcinoma and quantitative evaluation of neoplastic lesions in ventral (on the **left**) and lateral (**right**) prostates after PGZ treatment.

PGZ Treatment	No. of Rats	Ventral				Lateral			
		Incidence of Carcinoma	% of Lesions in Prostate ^a^			Incidence of Carcinoma	% of Lesions in Prostate ^a^		
			LG-PIN	HG-PIN	Carcinoma		LG-PIN	HG-PIN	Carcinoma
Control	10	10 (100%)	4.7 ± 1.9	80.7 ± 3.9	14.6 ± 3.7	8 (80%)	18.6 ± 7.9	78.6 ± 10.0	2.7 ± 3.8
1 mg/kg	8	8 (100%)	7.5 ± 2.2	83.3 ± 2.6	9.2 ± 2.8 **	5 (63%)	28.2 ± 15.8	70.7 ±16.3	1.0 ± 1.0
5 mg/kg	9	9 (100%)	10.4 ± 3.6 ***	81.9 ± 4.2	7.7 ±3.3 ***	4 (44%)	25.9 ± 8.6	73.3 ± 8.7	0.8 ± 0.8

^a^ Values expressed as the mean ± SD; ** *p* < 0.01, *** *p* < 0.001 compared to the control group; PGZ, pioglitazone; LG-PIN, low grade prostatic intraepithelial neoplasia; HG-PIN, high grade prostatic intraepithelial neoplasia.

## Supplementary Materials

Supplementary materials can be found at www.mdpi.com/1422-0067/17/12/2071/s1.
