# Supplementary material for: Pioglitazone, a Peroxisome Proliferator-Activated Receptor γ Agonist, Suppresses Rat Prostate Carcinogenesis
Source: Int J Mol Sci. 2016 Dec 10;17(12):2071. doi: 10.3390/ijms17122071 (PMC5187871; doi:10.3390/ijms17122071)
Supplement: Supplementary file 1 [file ijms-17-02071-s001.pdf]

Supplementary Materials: Pioglitazone, a Peroxisome Proliferator-Activated Receptor γ Agonist, Suppresses Rat Prostate Carcinogenesis

Shugo Suzuki, Yukiko Mori, Aya Nagano, Aya Naiki-Ito, Hiroyuki Kato, Yuko Nagayasu, Mizuho Kobayashi, Toshiya Kuno and Satoru Takahashi

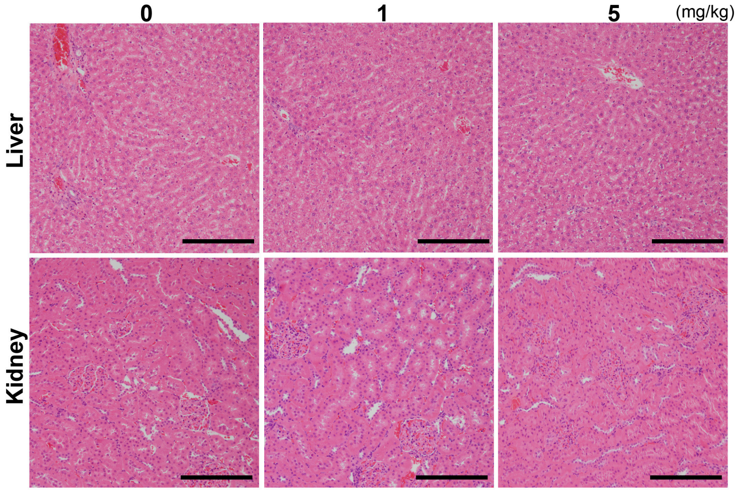

Figure S1. Histology of liver and kidney. Representative histopathological findings for lesions in the liver and kidney of the 0 (control), 1 and 5 mg/kg PGZ-treated groups. Scale bars = 200 μm.

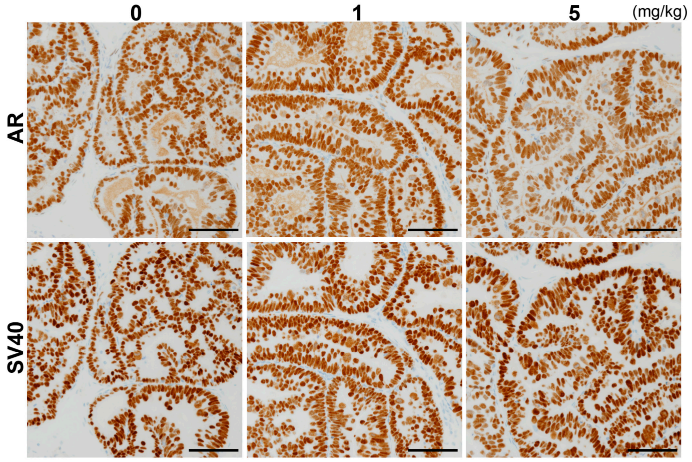

Figure S2. Immunohistochemistry of AR and SV40. Representative immunohistochemistry of AR and SV40 in the ventral prostate of the 0 (control), 1 and 5 mg/kg PGZ-treated groups. Scale bars = 50 μm.
